# Coarticulatory vowel nasalization in American English: Data of individual differences in acoustic realization of vowel nasalization as a function of prosodic prominence and boundary

**DOI:** 10.1016/j.dib.2019.104593

**Published:** 2019-10-01

**Authors:** Daejin Kim, Sahyang Kim

**Affiliations:** a Department of Linguistics, University of New Mexico, Albuquerque, NM, USA; b Department of English Education, Hongik University, Seoul, South Korea

**Keywords:** Vowel nasalization, Coarticulation, Prosodic strengthening, Prominence, Boundary, Phonetic grammar

## Abstract

This article provides acoustic measurements data for vowel nasalization which are based on speech recorded from fifteen (8 female and 7 male) native speakers of American English in a laboratory setting. Each individual speaker's production patterns for the vowel nasalization in tautosyllabic CVN and NVC words are documented in terms of three acoustic parameters: the duration of nasal consonant (N-Duration), the duration of vowel (V-Duration) and the difference between the amplitude of the first formant (A1) and the first nasal peak (P0) obtained from the vowel (A1-P0) as an indication of the degree of vowel nasalization. The A1-P0 is measured at three different time points within the vowel –i.e., the near point (25%), midpoint (50%), and distant point (75%), either from the onset (CVN) or the offset (NVC) of the nasal consonant. These measures are taken from the target words in various prosodic prominence and boundary contexts: phonologically focused (PhonFOC) vs. lexically focused (LexFOC) vs. unfocused (NoFOC) conditions; phrase-edge (i.e., phrase-final for CVN and phrase-initial for NVC) vs. phrase-medial conditions. The data also contain a CSV file with each speaker's mean values of the N-Duration, V-Duration, and A1-P0 (*z*-scored) for each prosodic context along with the information about the speakers' gender. For further discussion of the data, please refer to the full-length article entitled “Prosodically-conditioned fine-tuning of coarticulatory vowel nasalization in English”(Cho et al., 2017).

**Table:** Specifications Table

Subject area	*Linguistics*
More specific subject area	*Phonetics*
Type of data	*Table, figure, spreadsheet, CSV file*
How data was acquired	*Acoustic measurements based on speech recorded in a laboratory setting*
Data format	*Raw data*
Experimental factors	*The durations of nasal consonants and vowels, and the degree of vowel nasalization within the vowel were measured for the nasal consonant (N) and the neighboring vowel (V) at the syllable-initial (NVC) position and at the syllable-final (CVN) position. Two main experimental factors were focus types (lexical focus vs. phonological focus vs. no focus) and boundary types (the presence vs. absence of Intonational Phrase boundary).*
Experimental features	*Preparation of the data involved acquisition of acoustic data and analyses of nasal duration, vowel duration, and vowel nasalization (A1-P0)*
Data source location	*Hanyang University, Seoul, Korea*
Data accessibility	*Data are within the article*
Related research article	Cho, T., Kim, D., & Kim, S. (2017). Prosodically-conditioned fine-tuning of coarticulatory vowel nasalization in English. *Journal of Phonetics*, *64*, 71–89 [1].

**Table:** 

**Value of the data** • The data illustrate fifteen individual American English speakers' speech patterns for the coarticulatory vowel nasalization in various prosodic contexts (prominence and boundary). • The data can be used to examine speaker variation and the gender-related differences (eight females, seven males) in the phonetic realization of vowel nasalization in different prosodic contexts. • The data can be used for future studies to further examine cross-language or cross-dialectal similarities and differences in prosodically-conditioned vowel nasalization. • The data will inform further studies of individual speech variation under the rubric of the phonetics-prosody interface. • The attached CSV file contains individual speakers' mean values of nasal consonant duration (N-duration), vowel duration (V-duration) and A1-P0 (the difference between the amplitude of the first formant (A1) and the first nasal peak (P0)) for each prosodic condition, which can be used to run additional statistical analyses.

## 1 Data

The data presented in this article illustrate fifteen American English speakers' individual patterns of the acoustic realizations of vowel nasalization in tautosyllabic CVN and NVC words in various prosodic prominence and boundary contexts, which are related to Ref. [1]. A supplementary CSV file is attached, which contains individual speakers' mean values of the acoustic nasal duration, vowel duration, and A1-P0 for each prosodic condition (See an example in Table 1).

**Table 1:** Part of the CSV file that illustrates the organization of the file with respect to experimental conditions. The file contains each speaker's mean value of N-Duration, V-Duration and A1-P0 for each prosodic condition (Prominence and Boundary). This table contains the sample data values from speaker F1 producing the word bomb (CVN#) and mob (#NVC) in three prominence and two boundary conditions.

Speaker ID	Context	Prominence	Boundary	Timepoint	N-Duration	V-Duration	A1-P0
F01	CVN# (*bomb*)	Phonologically Focused (PhonFOC)	IP-final	75%	160.31	310.18	1.202
				50%	160.31	310.18	0.926
				25%	160.31	310.18	−0.600
			IP-medial	75%	149.26	224.06	0.854
				50%	149.26	224.06	0.061
				25%	149.26	224.06	0.046
		Lexically Focused (LexFOC)	IP-final	75%	67.97	254.07	1.436
				50%	67.97	254.07	−0.594
				25%	67.97	254.07	−0.495
			IP-medial	75%	121.43	204.6	1.118
				50%	121.43	204.6	−0.710
				25%	121.43	204.6	0.171
		Unfocused (NoFOC)	IP-final	75%	124.01	208.12	−0.358
				50%	124.01	208.12	−1.797
				25%	124.01	208.12	−1.085
			IP-medial	75%	63.08	158.12	0.138
				50%	63.08	158.12	−1.046
				25%	63.08	158.12	−1.164
	#NVC (*mob*)	Phonologically Focused (PhonFOC)	IP-initial	25%	71.34	234.18	1.375
				50%	71.34	234.18	1.692
				75%	71.34	234.18	1.134
			IP-medial	25%	137.96	218.11	0.162
				50%	137.96	218.11	0.905
				75%	137.96	218.11	0.931
		Lexically Focused (LexFOC)	IP-initial	25%	103.26	208.33	1.019
				50%	103.26	208.33	1.286
				75%	103.26	208.33	0.989
			IP-medial	25%	142.87	226.57	0.791
				50%	142.87	226.57	1.271
				75%	142.87	226.57	1.209
		Unfocused (NoFOC)	IP-initial	25%	38.33	182.38	0.384
				50%	38.33	182.38	0.948
				75%	38.33	182.38	1.268
			IP-medial	25%	78.85	149	0.169
				50%	78.85	149	−0.029
				75%	78.85	149	0.282

**Table 2:** List of target words and words that are contrasted phonologically (PhonFOC) and lexically (LexFOC).

CVN words			NVC words		
Target words	PhonFOC (phonologically contrasting words)	LexFOC (semantically contrasting words)	Target words	PhonFOC (phonologically contrasting words)	LexFOC (semantically contrasting words)
*pal****m***	*po****p***	*foot*	***m****op*	*bop*	*wash*
*bo****mb***	*Bo****b***	*war*	***m****ob*	*Bob*	*gang*
*te****n***	*Te****d***	*five*	***n****et*	*debt*	*ball*
*de****n***	*deb****t***	*cave*	***N****ed*	*dead*	*Paul*

### 1.1 The coarticulatory vowel nasalization in CVN words

**Fig. 1:**
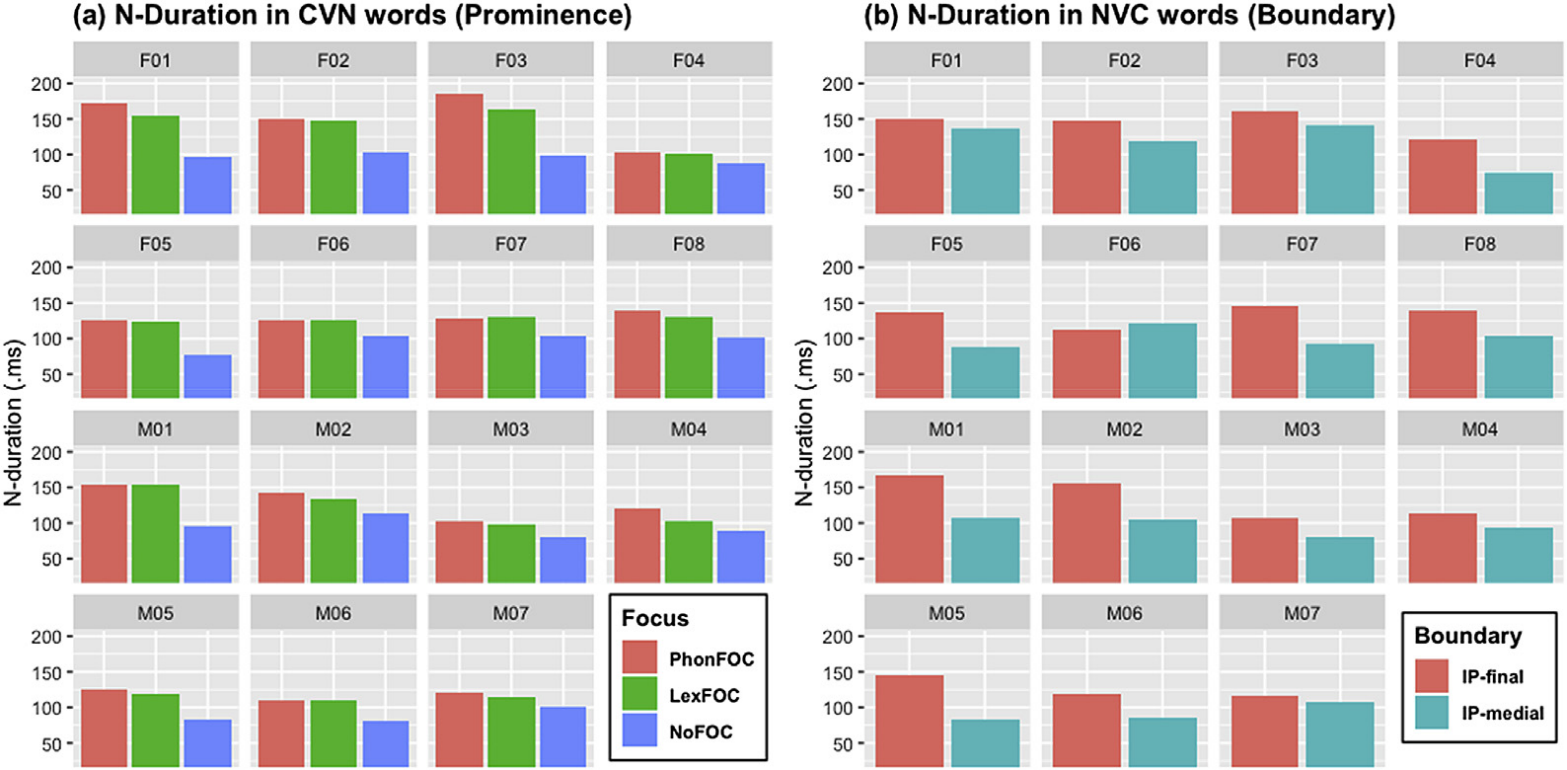
Barplots for the duration of nasal consonant (N-Duration) in the CVN words across 15 speakers as a function of (a) prosodic prominence (PhonFOC vs. LexFOC vs. NoFOC conditions) and (b) prosodic boundary (IP-final vs. IP-medial conditions).

**Fig. 2:**
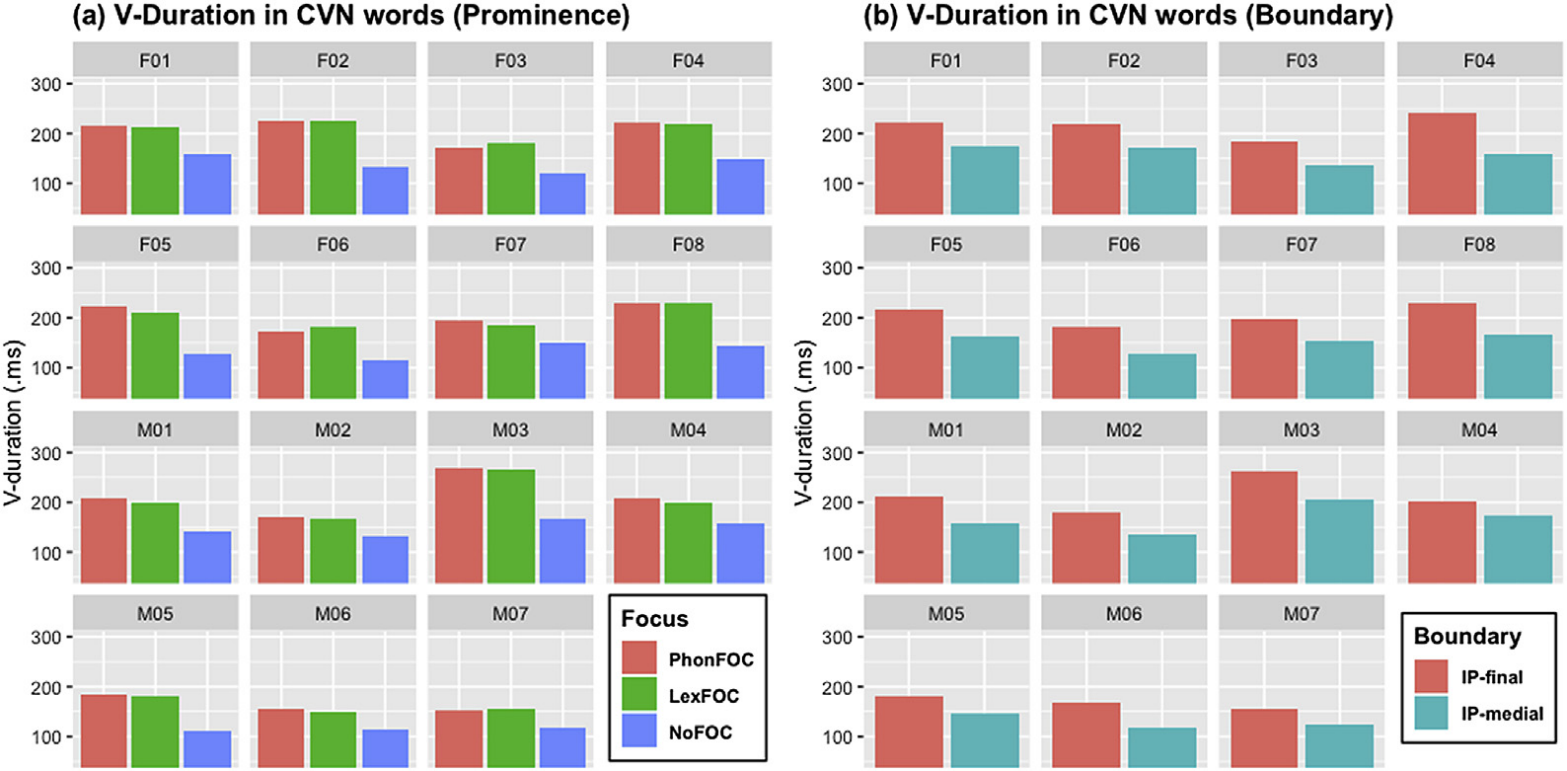
Barplots for the duration of vowel (V-Duration) in the CVN words across 15 speakers as a function of (a) prosodic prominence (PhonFOC vs. LexFOC vs. NoFOC conditions) and (b) prosodic boundary (IP-final vs. IP-medial conditions).

**Fig. 3:**
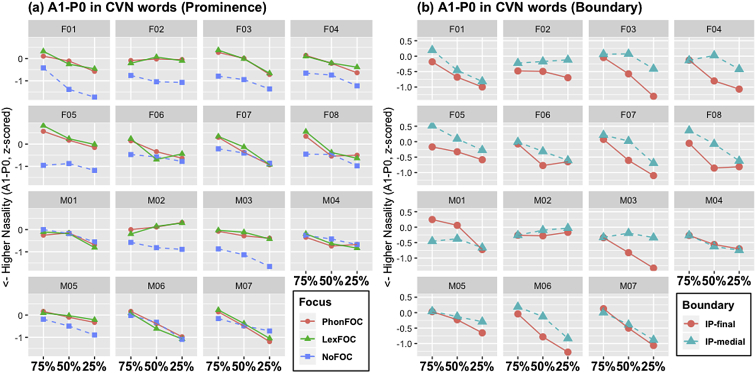
Line-point plots for the A1-P0 trends for the CVN words across three timepoints within the vowel. (Note that the 25% point is the nearest timepoint from the nasal consonant in CVN.) Data from fifteen speakers presented as a function of (a) prosodic prominence (PhonFOC vs. LexFOC vs. NoFOC conditions) and (b) prosodic boundary (IP-final vs. IP-medial conditions).

Fig. 1, Fig. 2, Fig. 3 (Fig. 1: N-duration; Fig. 2: V-duration; Fig. 3: A1-P0) show how each individual speaker's production of anticipatory vowel nasalization in CVN words changes as a function of prosodic prominence induced by focus (PhonFOC vs. LexFOC vs. NoFOC) and prosodic boundary (IP-final vs. IP-medial). As for the three prosodic prominence conditions, PhonFOC indicates that a target nasal consonant in CVN received focus by being phonemically contrasted with an oral coda consonant in a corresponding CVC word (e.g., ‘*bomb’* [bɑ**m**] vs. ‘*bob’* [bɑ**b**]); LexFOC indicates that a target CVN word received focus by being lexically contrasted with a semantically related word (e.g., ‘*bomb’* vs. ‘*war’*); and NoFOC indicates the absence of phonemic or lexical focus on a target CVN word. The word-final nasal consonant in CVN words appears at the end of an Intonational Phrase (IP-final) or in the middle of an Intonational Phrase (IP-medial).

The acoustic duration of nasal consonant which is the source of nasalization (N-duration), and that of vowel (V-duration) are illustrated in Fig. 1, Fig. 2, respectively. The degree of vowel nasalization, as indicated by the z-scored A1-P0 (i.e., the difference between the amplitude of the first formant (A1) and the first nasal peak (P0)), is shown in Fig. 3. Lower A1-P0 values indicate a *higher* degree of vowel nasalization. The A1-P0 is taken from three time points within the vowel – i.e., the near point (25%), midpoint (50%), and distant point (75%) from the nasal onset in the CVN sequence. Within each figure, (a) provides the data in three prominence conditions and (b) in two boundary conditions. The speaker gender (F for female and M for male) and ID number is presented on the top of each graph.

### 1.2 The coarticulatory vowel nasalization in NVC words

**Fig. 4:**
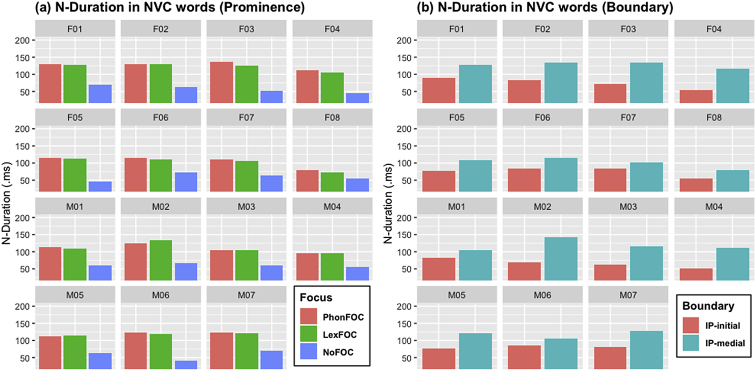
Barplots for the duration of nasal consonant (N-Duration) in the NVC words across 15 speakers as a function of (a) prosodic prominence (PhonFOC vs. LexFOC vs. NoFOC conditions) and (b) prosodic boundary (IP-initial vs. IP-medial conditions).

**Fig. 5:**
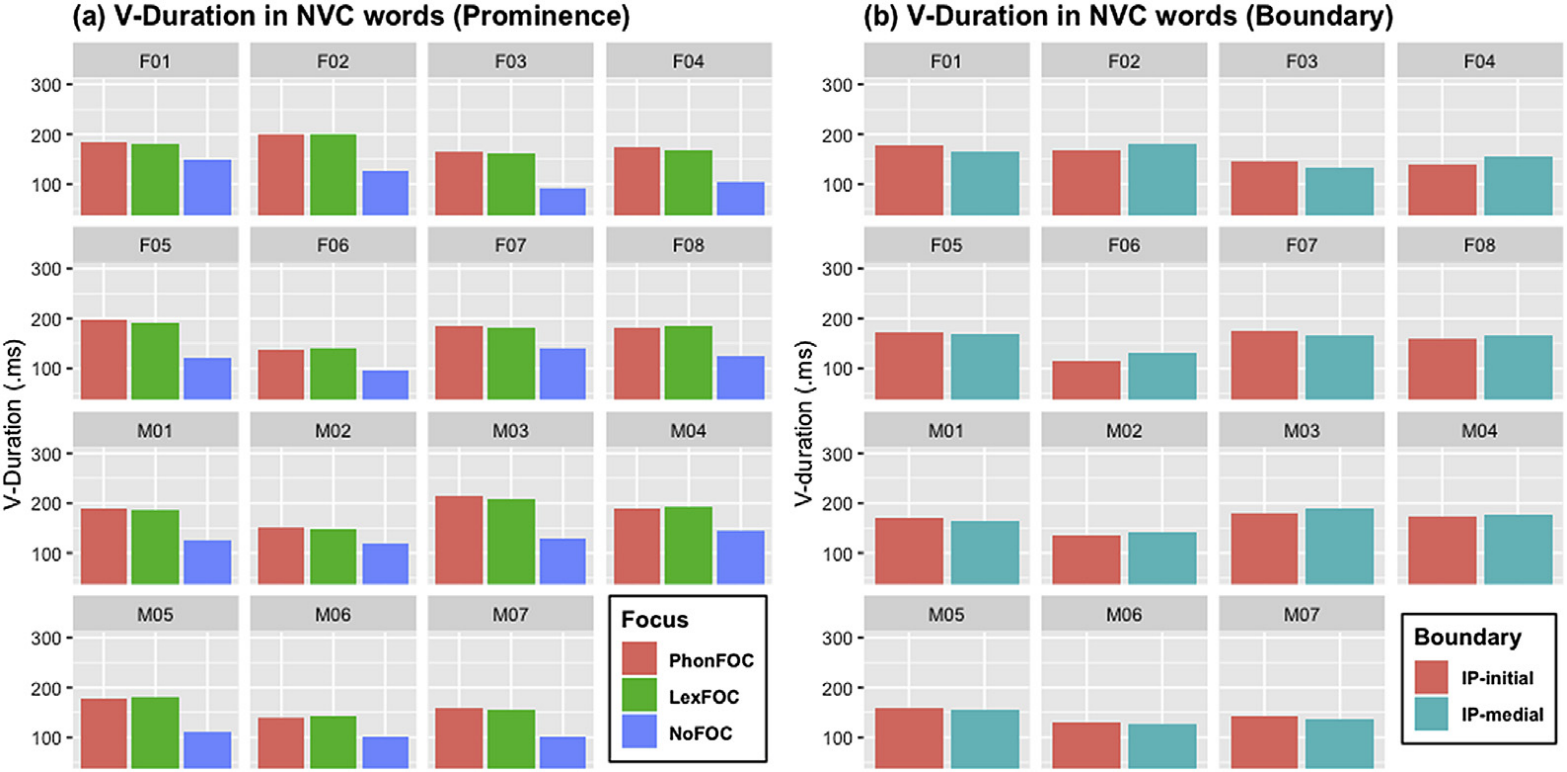
Barplots for the duration of vowel (V-Duration) in the NVC words across 15 speakers as a function of (a) prosodic prominence (PhonFOC vs. LexFOC vs. NoFOC conditions) and (b) prosodic boundary (IP-initial vs. IP-medial conditions).

**Fig. 6:**
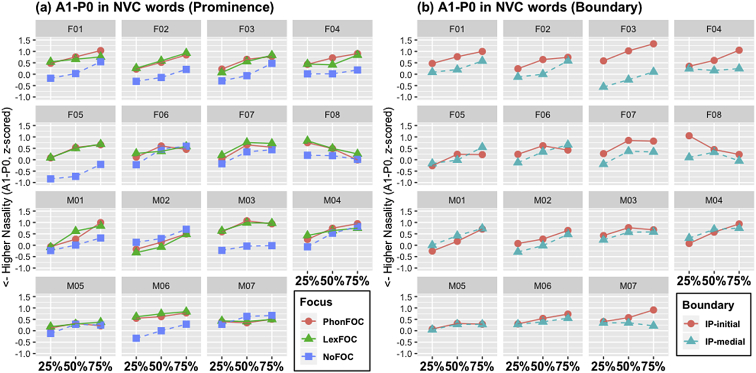
Line-point plots for the A1-P0 trends for the NVC words across three timepoints within the vowel. Note that the 25% point is the nearest timepoint from the nasal consonant in NVC. Data from fifteen speakers presented as a function of (a) prosodic prominence (PhonFOC vs. LexFOC vs. NoFOC conditions) and (b) prosodic boundary (IP-final vs. IP-medial conditions).

Fig. 4, Fig. 5, Fig. 6 (Fig. 4: N-duration; Fig. 5: V-duration; Fig. 6: A1-P0) illustrate how the fifteen individual speakers of American English produce the carryover vowel nasalization when the nasal consonant precedes a vowel in NVC words. As in CVN words, each figure shows how the three acoustic measures change as a function of prosodic prominence and boundary. Three prosodic prominence conditions are PhonFOC with a phonological contrast, LexFOC with a word meaning contrast, and NoFOC with no focus on the target word, just the same as in CVN words in 1.1. The prosodic boundary conditions for NVC words, however, differed from those for CVN words as the word-initial nasal consonant in NVC words appeared either at the beginning or in the middle of an Intonational Phrase, hence IP-initial and IP-medial boundary conditions.

Fig. 4, Fig. 5 provide individual speakers' data in terms of nasal duration (N-duration) and vowel duration (V-duration), respectively. Fig. 6 illustrates the degree of acoustic vowel nasalization (A1-P0) by individual speakers. The A1-P0 is taken from three time points within the vowel – i.e., the near point (25%), midpoint (50%), and distant point (75%) from the nasal offset in the NVC sequence, with lower values indicating higher nasality. Note that Fig. 3, Fig. 6 show a mirror image in terms of the direction of three time points within the vowel. In Fig. 3 for CVN words, the end of the vowel is closer to the nasal consonant, the source of nasalization; in Fig. 6 for NVC words, the beginning of the vowel is closer to the nasal consonant. Again, within each figure, (a) provides the data in three prominence conditions and (b) in two boundary conditions, with the speaker gender and ID number on the top of each graph.

### 1.3 Individual speakers' mean values for each condition

The CSV file attached to this article contains each individual speaker's mean values of N-duration, V-duration, and A1-P0 (z-scored) in different prosodic conditions. The organization of the file in terms of experimental factors is illustrated in Table 1. Each speaker is labeled with ‘F’ (female) or ‘M’ (male) and the ID number. There are three experimental factors of Context (CVN# vs. #NVC), Prominence (PhonFOC vs. LexFOC vs. NoFOC), and Boundary (IP-final vs. IP-medial for CVN#; IP-initial vs. IP-medial for #NVC). Timepoint (25%, 50%, 75% within a vowel) indicates a relative timepoint during a vowel at which A1-P0 values are taken. This file can be used for carrying out further statistical analyses of the data.

## 2 Experimental design, materials and methods

### 2.1 Participants

Eight female and seven male native speakers of American English in their 20s and early 30s were paid to participate in the recording. They were from either the Midwest or the West Coast of the United States. All of them resided temporarily in Korea as exchange students or English teachers at the time of the recording.

### 2.2 Speech materials for acoustic recordings

There were four CVN and four NVC target words. CVN words had an oral stop onset and a nasal stop (/m/or/n/) coda, and NVC words had a nasal stop (/m/or/n/) onset and an oral stop coda. The vowel was either/ɑ/or/ɛ/in both CVN and NVC words.

Each target word was produced in six carrier sentences with different prosodic structure (i.e., 3 prominence x 2 boundary conditions). As shown in Table 3, a carrier sentence with a target word was part of a mini dialogue consisted of a question and an answer and it was always an answer (B's in Table 3) to a prompt question (A's in Table 3). The mini dialogues were created so that the speakers would produce the target words produced in various prosodic conditions. As shown in Table 3a, a contrastive focus context was employed to induce focus (via a nuclear pitch accent) on the test word. A test word *bomb* in an answer (B) was contrasted with a word in a prompt sentence (A) either phonologically (‘*Bob’*) or lexically (‘*war’*) in focus conditions. When the target word was in No Focus condition, the focus fell elsewhere in the answer as in Table 3b, d, f, h such that the target word, already given in a question, was naturally unaccented. As for the boundary conditions, the CVN target words (e.g., ‘*bomb*’) occurred either in the Intonational Phrase final position (i.e., IP-final) as in Table 3a, b or in the Intonation Phrase medial position (i.e., IP-medial) as in Table 3c, d. Note that in the IP-medial conditions, the target words were produced in the middle of a short quoted phrase (e.g., ‘*say BOMB fast again*’) so that no prosodic boundary would be inserted before and after the target words. The NVC target words (e.g., ‘mob’) occurred either in the Intonational Phrase initial position (i.e., IP-initial) as in Table 3 e, f or in the Intonational Phrase medial position (i.e., IP-medial) as in Table 3 g, h.

**Table 3:** The target words ‘*bomb’* and *‘mob’* produced in carrier sentences with two Boundary conditions (IP-final for *bomb* and IP-initial for *mob* vs. IP-medial) and three Focus conditions (PhonFOC vs. LexFOC vs. NoFOC). The focused words are marked in bold, and the test word is underlined.

a. **IP-final, PhonFOC (LexFOC) (where ‘*#*’ = IP boundary)** A: *Were you supposed to write **BOB** (**WAR**)?* B: *No. I was supposed to write **BOMB** #, wasn't I?*
b. **IP-final, NoFOC (where ‘#’ = IP boundary)** A: *Were* ***YOU*** *supposed to write bomb?* B: *No.* ***JOHN*** *was supposed to write bomb #, wasn't he?*
c. **IP-medial, PhonFOC (LexFOC)** A: *Did you write ‘say* ***BOB*** *(****WAR****) fast again’?* B: *No. I wrote ‘say #* ***BOMB*** *fast again’.*
d. **IP-medial, NoFOC** A: *Did you write ‘say bomb* ***FAST*** *again’?* B: *No. I wrote ‘say bomb* ***SLOWLY*** *again.*
e. **IP-initial, PhonFOC (LexFOC) (where ‘***#***’ = IP boundary)** A: *Did you write ‘****BOB*** *(****GANG****) fast again’?* B: *Not exactly. # ‘****MOB*** *fast again’ was what I wrote.*
f. **IP-initial, NoFOC (where ‘*#*’ = IP boundary)** A: *Did you write ‘mob* ***FAST*** *again’?* B: *Not exactly. # ‘Mob* ***SLOWLY*** *again’ was what I wrote.*
g. **IP-medial, PhonFOC (LexFOC)** A: *Did you write ‘say* ***BOB*** *(****GANG****) fast again’?* B: *No. I wrote ‘say #* ***MOB*** *fast again’.*
h. **IP-medial, NoFOC** A: *Did you write ‘say mob* ***FAST*** *again’?* B: *No. I wrote ‘say mob* ***SLOWLY*** *again.*

Prompt sentences were pre-recorded by a female native speaker of American English. During the data collection, a participant sat in front of a PC, heard a prompt question (A's in Table 3) through a speaker and saw it visually presented on the monitor. The participant then answered the question by reading a corresponding target-bearing sentence (B's in Table 3) presented visually on the monitor.

The recordings took place in a sound-attenuated booth at Hanyang Phonetics and Psycholinguistics Lab at a sampling rate of 44 kHz using a SHURE KSN 44 dynamic microphone and a Tascam HD-P2 digital recorder. Sentences were presented on a computer screen in a randomized order and repeated four times across four blocks. Speakers were asked to listen to the prime questions and to answer them by reading the target sentences aloud with the meaning contrast in mind. At the time of recording, when the experimenter, a trained prosody transcriber, noticed any production error, he asked the speaker to read the sentence a few more times to obtain utterances produced as naturally as possible. Each recording session took about 70–90 minutes, including three 5-min breaks. A total of 2880 tokens were collected: 2 boundary conditions (IP-initial for #NVC/IP-final for CVN# vs. IP-medial) x 3 focus conditions (PhonFOC vs. LexFOC vs. NoFOC) x 8 target words (as in Table 2) x 4 repetitions x 15 speakers. Two trained phoneticians reviewed all the data collected to check if they were produced with intended prosodic renditions in terms of prominence and boundary. When the tokens were produced with unintended accent placement or boundary, they were excluded from further analyses. As a result, 321 tokens were discarded, leaving 2786 tokens for acoustic analyses.

### 2.3 Measurements

The following acoustic measures were taken from a nasal stop and a vowel in the CVN and NVC words, using Praat [2].

#### 2.3.1 Nasal (N-Duration) and vowel (V-Duration) Durations

N-duration is the duration of the nasal consonant, taken from the onset to the offset of the nasal energy (murmur) and nasal zeros (weakened formant structure) displayed on the spectrogram. V-duration is the duration of the vowel, measured from the beginning to the end of the vowel's period complex waveform. The vowel's waveform was cross-checked with the vowel's formant structure displayed on the spectrogram.

#### 2.3.2 The degree of vowel nasalization (A1-P0)

The nasal murmur is identified near the first formant (F1), which decreases the amplitude of F1 (i.e., A1) and increases the amplitude of the nasal peak (i.e., P0) around the fundamental frequency. The lower the A1-P0, therefore, the more the vowel is nasalized (see Fig. 1 in Ref. [1] for the graphic explanation) [3]. To observe how the degree of vowel nasalization changes as a function of time in various prosodic contexts, the A1-P0 was measured at three time points within the vowel – i.e., the near point (at the 25% point of the vowel duration from the nasal consonant), the midpoint (at the 50% point of the vowel duration), and the distant point (at the 75% point of the vowel away from the nasal consonant). The A1-P0 was measured by a Praat script provided by W. Styler and R. Scarborough [4]. Some measured values were discarded (i) when the amplitudes of the first and second harmonics calculated were erroneously similar, (ii) when the pitch was erroneously detected at less than 85Hz or more than 300Hz, and (iii) when the Praat script itself failed to find an accurate value of pitch and harmonic structure [4]. This procedure removed 385 data points out of 8348 data points measured, leaving the total of 7963 A1-P0 values for the analyses. The A1-P0 values were further standardized (*z*-scored) within each speaker to minimize the individual variances across speakers.
